# External validation of outcome prediction scores for aneurysmal subarachnoid hemorrhage in a real-world setting: a monocentric experience

**DOI:** 10.1007/s10072-025-08232-5

**Published:** 2025-05-17

**Authors:** Raffaele De Marco, Riccardo Melilli, Riccardo Russo, Mauro Bergui, Diego Garbossa, Fabio Cofano

**Affiliations:** 1 Department of Neuroscience “Rita Levi Montalcini”, University of Turin, Via Cherasco, 15, 10126 Turin, Italy; 2 Neurosurgery Unit, “Città della Salute e della Scienza” University Hospital, Turin, Italy; 3 Interventional Neuroradiology Unit, “Città della Salute e della Scienza” University Hospital, Turin, Italy

**Keywords:** Cerebral aneurysm, Subarachnoid hemorrhage, Prognostic score

## Abstract

**Objective:**

To evaluate the applicability and performance of predictive scoring systems for outcome prediction in a real-world cohort of patients with aneurysmatic Subarachnoid Hemorrhage (aSAH), trying to avoid overtreatment and reducing their impact on Intensive Care Unit (ICU) overcrowding.

**Methods:**

All adult patients diagnosed with aSAH from January 2018 to December 2023 were retrospectively collected in a single centre. Predictive scores such as SAFIRE, HATCH, HAIR, and Nutshell were calculated, and their performance was analyzed using receiver operating characteristic (ROC) analysis. Kaplan-Meier curves were estimated by dividing the cohort according to the calculated scores.

**Results:**

Of 274 patients, 72.3% underwent endovascular treatment, 16.1% surgical clipping, and 11.7% received no treatment. The 30-day and overall mortality rates were 27.7% and 41.2%, respectively. ICU admission occurred in 74.1% of cases, with a mortality rate of 38.4%. The HAIR and the Nutshell scores performed well in predicting 30-day mortality (AUC: 0.812 and 0.749, respectively), while the SAFIRE and the HATCH scores demonstrated excellent predictive value for poor functional outcomes (AUC: 0.866 and 0.886 at discharge; 0.825 and 0.83 at one year, respectively). Patients in the worst SAFIRE group (> 15) exhibited mortality rates exceeding 89% at follow-up, with universally poor outcomes.

**Conclusion:**

The SAFIRE and the HATCH scores are reliable tools for predicting outcomes in aSAH patients, with SAFIRE offering particular value at admission. Their implementation in surgical practice could improve resource allocation, guide ethical decision-making, and reduce ICU burden, especially for patients with severe aSAH and limited potential for recovery.

**Supplementary Information:**

The online version contains supplementary material available at 10.1007/s10072-025-08232-5.

## Introduction

Although aneurysmal subarachnoid hemorrhage (aSAH) accounts for only 5% of all strokes, it has a notable detrimental effect on the social and economic burden, affecting primarily young adults in their working age [1–3]. Mortality rates are high (reaching 40%), but the greatest impact is on survivors, who often lack the capacity for independent daily activities, reflecting a significant commitment to caregivers. The potential years of life lost due to aSAH have an impact similar to that produced by ischemic stroke and intracerebral hemorrhage [4]. Despite progress in resuscitation management, remote outcomes are still poor (mostly depending on the initial clinical status), as 25% of survivors are not independent at 3 months, and more than 30% report low long-term quality of life [5, 6]. For this reason, a better selection of patient who can really benefit from treatment could be useful in terms of cost for the health system and caregivers at discharge. Numerous prognostic scores have been developed to estimate 30-day mortality and the degree of disability in the short- and long-term periods. Firstly, these tools are designed to assist physicians in communicating with patients’ families and providing guidance on ethically challenging decisions. Secondly, they aim to ensure the rational allocation of limited resources to patients who require them most [7]. However, such scores are not yet applied in clinical practice, in part for ethical reasons. This analysis aims to evaluate retrospectively their applicability, performance, and validation in a “real-world” setting analyzing the possible reduction in ICU overcrowding and overtreatment for those patients who would not get a real clinical benefit.

## Methods

All the nontraumatic subarachnoid hemorrhages (SAH) (ICD-9-CM 430) diagnosed at the authors’ institution in the period January 2018– December 2023 were retrospectively scanned. The analysis included adult patients (≥ 18 years old) with a radiologically confirmed aneurysmal subarachnoid hemorrhage (aSAH). Only saccular or fusiform aneurysmatic malformations were considered for inclusion. In contrast, the absence of an aneurysmatic malformation or other causes of SAH at two consecutive digital subtraction angiographies (DSA) - defining the picture of unknown SAH or *“sine materia”* - was considered an exclusion criterion. Since there was no interaction or intervention with the subject, and in accordance with institutional policy, it was deemed sufficient to waive the requirement for patient consent. Moreover, the study was of a retrospective nature, thus rendering it unnecessary to obtain approval from an institutional review board.

Demographic data and patient-specific risk factors such as smoking status (specifically active smoking), essential hypertension, dyslipidemia, coronary artery disease, diabetes mellitus type 2, pathological obesity (body mass index > 29.9), use of drugs, or previous stroke were retrieved from clinical records. Familiar history of cerebrovascular diseases (aneurysmatic rupture and other cerebrovascular accidents) was collected as well. SAH severity was calculated at emergency department admission using the Glasgow Coma Scale (GCS), the Hunt and Hess (H&H) scale and the World Federation of Neurological Surgeon (WFNS) grading system.

Other information and classifications were defined based on computed tomography angiography (CTA) images whose availability was considered for inclusion in the study: specifically, the thickness of the SAH (measured in millimeters (mm) and perpendicular to the axial plan in the cistern that contained the largest quantity of blood) and blood in the ventricular system were considered for calculation of modified Fisher Scale (mFS) and the Barrow Neurological Institute (BNI) grading scale. Aneurysm’s multiplicity and ruptured aneurysm characteristics, such as location (including both anterior and posterior circulation aneurysm) and size, were obtained from CTA and DSA from trained neuroradiologists. In the event that any procedure was performed on the patient prior to the treatment of the aneurysm (whether surgical - such as an external ventricular drain - or not), the resuscitated Glasgow Coma Scale (GCS) (after treatment) was considered as a reference.

Patients were assigned to different groups considering the treatment they received for securing the aneurysm: no treatment, surgical treatment, or endovascular treatment (e.g., coiling, flow diverter, or both). A distinction between intensive care (ICU) and ward was made in the setting after treatment or in case of no treatment. Length of stay in both settings was recorded as well as the complications. Among the latter both cerebral ones directly connected to the SAH, such as hydrocephalus, cerebral vasospasm, delayed cerebral ischemia, rebleeding, and systemic complications such as infections were considered for each patient during his/her stay in the ICU or ward. Death event was distinguished by setting (death in the ICU, ward, or generally in the hospital) and time, as the percent risks of death at 30 days, 60 days, and overall.

If the patient survived, the length of the in-hospital stay, discharge disposition, and outcome measures, such as the Glasgow Outcome Scale (GOS) and the modified Rankin Scale (mRS) were reported. A minimum follow-up of 6 months was considered for outcome evaluation. GOS and mRS were again evaluated at the last available follow-up visit. GOS and mRS were dichotomized in favorable and unfavorable outcomes considering for GOS an unfavorable outcome if ≤ 3 while for mRS if ≥ 3.

### Predictive outcome scores

The collection of demographic variables allowed the possibility to retrospectively calculate and validate different scores in a population whose decision-making process for management of the aSAH was defined on a case-by-case basis in a multidisciplinary context and by assessing the complexity of factors that sometimes goes beyond those collected in databases: the SAFIRE score [8] and the HATCH (Hemorrhage, Age, Treatment, Clinical State, Hydrocephalus) Score [9].

Five different groups were identified for the SAFIRE score (1 = 0–2; 2 = 3–5; 3 = 6–8; 4 = 9–15; 5 = > 15) and four for the HATCH score (1 = 0–4; 2 = 5–6; 3 = 7–8; 4 = 9–13). Two other different scores [with the type of treatment among variables, e.g. Nutshell-Tool (Nijmegen acute subarachnoid hemorrhage calculator), an artificial intelligence (AI)-based tool [10], and without the treatment among variables for the score, such as the HAIR score [11])] were considered for comparison. Specifically, the different probabilities that Nutshell tool returns (i.e., the occurrence of in-hospital mortality (1), the occurrence of mRS > 2 at 6 months (2), the occurrence of DCI (3)) were reported separately.

### Statistical analysis

A statistical analysis was conducted using an open-source software built on R language (The jamovi project (2021). jamovi. (Version 2.6) [Computer Software]. Retrieved from https://www.jamovi.org). Nominal and Categorical variables were analyzed using Pearson’s Chi-square test, Trend test for ordinal variables, and/or the Kruskal Wallis. A Wilcoxon or a Fisher’s exact test or a t-student’s test were used for numerical variables.

The performance of the existing score was analyzed for the population in the study and a graphical representation was created through the Receiver Operating Characteristic (ROC). A De Long’s test was used for comparison between scores. Hazard ratio (HR) and survival rate with Kaplan-Meier estimation were evaluated as well. The level of statistical significance was set at *p* < 0.05.

## Results

The initial research retrieved 376 patients, who became 274 after excluding 52 patients with unknown SAH origin, 23 with spontaneous SAH due to amyloid angiopathy, 19 affected by arteriovenous malformations and 8 patients with mycotic aneurysms.

The mean age of the study population was 60.1 years old (±13.6; range 18.0–91.0) with a F: M ratio of almost 2:1. 36.5% of patients did not show any risk factor while another 35% had at least one risk factor. Hypertension was the most frequent one with 48.5% of patients. 22.6% was an active smoker. Positive familiar history for cerebrovascular events was not so frequent (6.6%) and 5.8% received treatment for a cerebral aneurysm in the past.

An aneurysm of the anterior circulation occurred in 83.9% compared to the 16.1% of cases located in the posterior circulation. Specifically, among the anterior circulation, the most common location was at the anterior cerebral arteries (ACA) complex (39.8%), followed by middle cerebral arteries (MCA) (20.8%), internal carotid arteries (ICA) (12.8%), and posterior communicating arteries (PComA) (10.6%) and.

34.3% had at least another aneurysm which had been diagnosed during initial work-up.

Considering the dome of the aneurysm, the mean size was 8.0 mm (±5.4; range 1.5–33.0).

Information regarding clinical and radiological scales is reported in supplementary Table 1.

Most patients (198/274, 72.3%) underwent endovascular treatment for securing the aneurysm (either coiling, flow diverter or both), while 44 (16.1%) underwent surgical clipping and 32 (11.7%) were judged not eligible for any treatment after multidisciplinary discussion. In this sense, age (*p* = 0.01), comorbidities (*p* = 0.05), aneurysm located in the posterior circulation (*p* = 0.01), high mFisher grade (*p* = 0.02), presence of intracerebral hemorrhage (*p* < 0.01), worst grade in all the clinical scales (GCS, H&H, WFNS) as well as the worst class in the SAFIRE score showed a significant relationship (*p* < 0.01) with the type of treatment.

An external ventricular drain was placed in 18.6% of cases and 74.1% were admitted in the ICU after securing the aneurysm. The mean LOS was 17.4 days (SD 19.6; range 1.0-108.00) and the mortality rate in ICU was 38.2%. A total of 186 patients were admitted to Neurosurgical ward and the mean LOS was 17.1 days (SD 14.0; range 1.0–83.0) with a mortality rate of 3.76%.

A total of 69.3% of the patients experienced at least one complication during the hospitalization (regardless of the setting). In ICU, 72 patients underwent tracheostomy; among 34 nosocomial pneumonia, 19 cases were recorded as Ventilator-Associated Pneumonia; other complications were 4 cases of meningitis and 4 cases of pulmonary embolism.

Considering only the cerebral ones, evidence of radiological vasospasm was diagnosed in 20.4% of cases of which only 13.9% were treated endovascularly (25 patients with balloon angioplasty, 6 with intra-arterial infusion of nimodipine– 3 of which in combination with the first procedure– and a stent was necessary in 2 cases), while delayed cerebral ischemia (DCI) was diagnosed in 15.0% of patients, and signs of chronic hydrocephalus were experienced by 19.7%. In our population, mFS, BNI grade, SAFIRE, and HATCH scores did not show a statistically significant relationship with the appearance of the radiological signs of vasospasm, but BNI grade (*p* = 0.01), SAFIRE (*p* = 0.03) and HATCH (*p* < 0.01) scores (as numerical variables) and the groups of the SAFIRE score (*p* < 0.01) reached statistical significance.

**Fig. 1:**
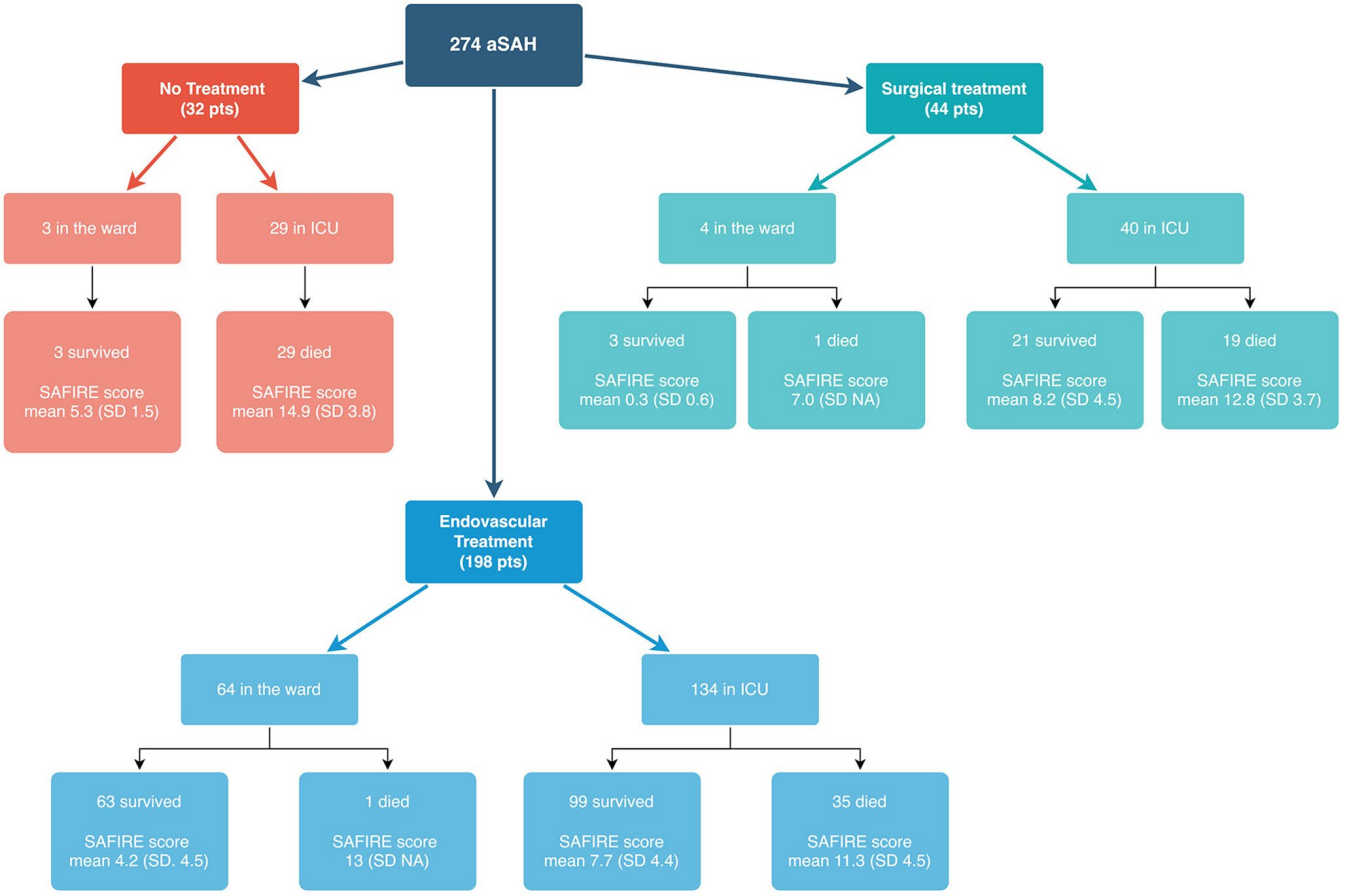
Variable Tree Diagram showing the different distribution of patients by treatments. Setting after treatment was reported (ward and ICU) and in the end the mortality in the hospital was differentiated for each treatment and setting reporting the mean (and standard deviation, SD) of the SAFIRE score

The mortality rate at 30 days was 27.7%, at 60 days was 31.4% and overall reached 41.2% considering a mean follow-up period of 18.1 months (SD 20.9, range 0–79). The rate of death in the hospital was 31.0% and differentiating by setting, 38.4% was the mortality rate in ICU and 3.6% in the ward (considering both patients admitted directly in the ward (*n* = 71) and those patients who did not die in ICU (*n* = 125)) (Fig. 1).

**Fig. 2:**
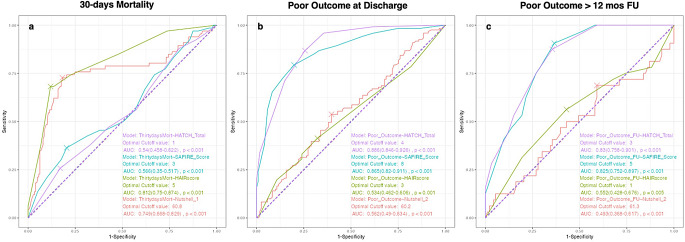
ROC analysis and comparison between scores in predicting 30-day mortality (**a**), poor outcome at discharge (**b**) and poor outcome in those patients with more than 12 months of follow-up (**c**). De Long’s test was applied for comparison

Age, sex, risk factors, clinical status at ED, radiological scales, type of treatment, SAFIRE and HATCH scores did not appear to influence the 30-day mortality rate (and similarly for 60-day mortality), while the HAIR score and the Nutshell (1) significantly differed in this comparison (Table 1). Indeed, an AUC of 0.812 and 0.749 was reached by HAIR score and for Nutshell (1), respectively, compared to 0.54 and 0.566 of the HATCH and the SAFIRE (Fig. 2a).

**Table 1:** 30- and 60-days mortality rate. Statistic tests: ^1^Linear model Anova; ^2^Pearson’s Chi-squared test; ^3^Trend test for ordinal variables

	30-days Mortality			60-days Mortality		
	No (*N* = 198)	Yes (*N* = 76)	*p* value	No (*N* = 188)	Yes (*N* = 86)	*p* value
**Age**			0.114^1^			**0.040** ^**1**^
Mean (SD)	60.9 (13.4)	58.0 (13.8)		61.2 (13.3)	57.6 (14.0)	
Range	18.0–91.0	23.0–89.0		18.0–91.0	23.0–89.0	
**Sex**			0.954^2^			0.619^2^
Female	131.0 (66.2%)	50.0 (65.8%)		126.0 (67.0%)	55.0 (64.0%)	
Male	67.0 (33.8%)	26.0 (34.2%)		62.0 (33.0%)	31.0 (36.0%)	
**Multiple Aneurysms**			0.149^2^			**0.040** ^**2**^
No	125.0 (63.1%)	55.0 (72.4%)		116.0 (61.7%)	64.0 (74.4%)	
Yes	73.0 (36.9%)	21.0 (27.6%)		72.0 (38.3%)	22.0 (25.6%)	
**Active Smoker**			0.699^2^			0.650^2^
No	152.0 (76.8%)	60.0 (78.9%)		144.0 (76.6%)	68.0 (79.1%)	
Yes	46.0 (23.2%)	16.0 (21.1%)		44.0 (23.4%)	18.0 (20.9%)	
**Essential Hypertension**			0.976^2^			0.475^2^
No	102.0 (51.5%)	39.0 (51.3%)		94.0 (50.0%)	47.0 (54.7%)	
Yes	96.0 (48.5%)	37.0 (48.7%)		94.0 (50.0%)	39.0 (45.3%)	
**Aneurysm Location**			0.869^3^			0.776^3^
ACA	77.0 (38.9%)	32.0 (42.1%)		74.0 (39.4%)	35.0 (40.7%)	
MCA	44.0 (22.2%)	13.0 (17.1%)		40.0 (21.3%)	17.0 (19.8%)	
PComA	20.0 (10.1%)	9.0 (11.8%)		19.0 (10.1%)	10.0 (11.6%)	
ICA	24.0 (12.1%)	11.0 (14.5%)		23.0 (12.2%)	12.0 (14.0%)	
Posterior Circulation	33.0 (16.7%)	11.0 (14.5%)		32.0 (17.0%)	12.0 (14.0%)	
**Aneurysm Size**			0.488^1^			0.686^1^
Mean (SD)	7.9 (5.4)	8.4 (5.4)		8.0 (5.4)	8.2 (5.3)	
Range	1.5–33.0	1.8–23.0		1.5–33.0	1.8–23.0	
**SAH Size**			0.758^1^			0.306^1^
Mean (SD)	8.0 (3.8)	8.2 (4.1)		7.9 (3.8)	8.4 (4.2)	
Range	0.0–19.8	0.0–20.0		0.0–17.0	0.0–20.0	
**mFisher Scale**			0.089^3^			0.112^3^
0	1.0 (0.5%)	1.0 (1.3%)		1.0 (0.5%)	1.0 (1.2%)	
1	13.0 (6.6%)	7.0 (9.2%)		13.0 (6.9%)	7.0 (8.1%)	
2	2.0 (1.0%)	0.0 (0.0%)		2.0 (1.1%)	0.0 (0.0%)	
3	48.0 (24.2%)	27.0 (35.5%)		43.0 (22.9%)	32.0 (37.2%)	
4	134.0 (67.7%)	41.0 (53.9%)		129.0 (68.6%)	46.0 (53.5%)	
**BNI Grade**			0.748^3^			0.833^3^
1	1.0 (0.5%)	1.0 (1.3%)		1.0 (0.5%)	1.0 (1.2%)	
2	45.0 (22.7%)	18.0 (23.7%)		44.0 (23.4%)	19.0 (22.1%)	
3	102.0 (51.5%)	40.0 (52.6%)		96.0 (51.1%)	46.0 (53.5%)	
4	38.0 (19.2%)	11.0 (14.5%)		37.0 (19.7%)	12.0 (14.0%)	
5	12.0 (6.1%)	6.0 (7.9%)		10.0 (5.3%)	8.0 (9.3%)	
**GCS**			0.207^3^			0.332^3^
**H&H**			0.314^3^			0.440^3^
**WFNS**			0.302^3^			0.400^3^
**Aneurysm Treatment**			0.851^3^			0.985^3^
No Treatment	22.0 (11.1%)	10.0 (13.2%)		21.0 (11.2%)	11.0 (12.8%)	
Clipping	35.0 (17.7%)	9.0 (11.8%)		32.0 (17.0%)	12.0 (14.0%)	
Coiling ± Flow Diverter	141.0 (71.2%)	57.0 (75.0%)		135.0 (71.8%)	63.0 (73.3%)	
**Complications during Hospitalization**			0.429^2^			0.858^2^
No	58.0 (29.3%)	26.0 (34.2%)		57.0 (30.3%)	27.0 (31.4%)	
Yes	140.0 (70.7%)	50.0 (65.8%)		131.0 (69.7%)	59.0 (68.6%)	
**SAFIRE Score**			0.266^1^			0.232^1^
Mean (SD)	8.6 (5.3)	7.8 (5.9)		8.7 (5.4)	7.8 (5.7)	
Range	0.0–22.0	0.0–22.0		0.0–22.0	0.0–22.0	
**SAFIRE Group**			0.253^3^			0.247^3^
1	30.0 (15.2%)	20.0 (26.3%)		29.0 (15.4%)	21.0 (24.4%)	
2	37.0 (18.7%)	12.0 (15.8%)		34.0 (18.1%)	15.0 (17.4%)	
3	35.0 (17.7%)	7.0 (9.2%)		33.0 (17.6%)	9.0 (10.5%)	
4	73.0 (36.9%)	29.0 (38.2%)		70.0 (37.2%)	32.0 (37.2%)	
5	23.0 (11.6%)	8.0 (10.5%)		22.0 (11.7%)	9.0 (10.5%)	
**HATCH Score**			0.370^1^			0.515^1^
N-Miss	22.0	10.0		21.0	11.0	
Mean (SD)	4.2 (2.5)	3.9 (2.4)		4.2 (2.5)	4.0 (2.5)	
Range	1.0–9.0	1.0–9.0		1.0–9.0	1.0–9.0	
**HAIR score**			**< 0.001** ^**1**^			**< 0.001** ^**1**^
Mean (SD)	1.8 (1.7)	4.4 (2.0)		1.7 (1.7)	4.3 (2.0)	
Range	0.0–7.0	0.0–7.0		0.0–7.0	0.0–7.0	
**Nutshell (1)**			**< 0.001** ^**1**^			**< 0.001** ^**1**^
Mean (SD)	36.9 (24.4)	65.2 (27.5)		35.7 (23.7)	64.5 (27.4)	
Range	2.0–99.7	3.8–100.0		2.0–99.7	3.8–100.0	

**Fig. 3:**
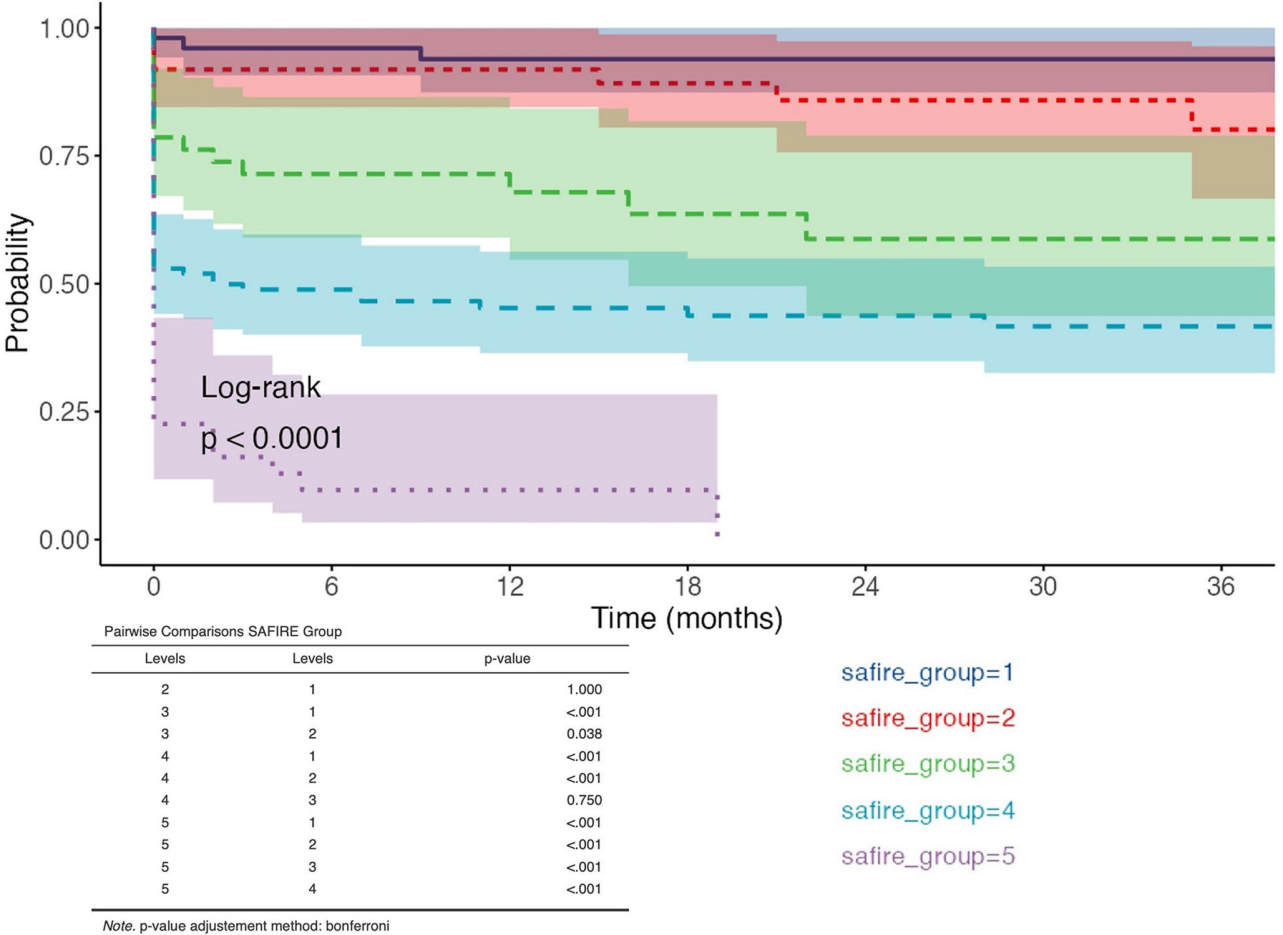
Survival analysis and Kaplan-Meier estimation differentiating by SAFIRE score and specific group. A pairwise comparison was reported (a Bonferroni adjustment was applied for p values)

Although these scores did not reach an optimal value of sensitivity and specificity in predicting 30-day and 60-day mortality rate, a significant difference exists between groups of the SAFIRE score analyzing Kaplan-Meier estimation of survival (Log-rank *p* < 0.0001) (Fig. 3). Specifically, in a pairwise comparison, the probability of being alive for group 5 patients was 16.1% (95%CI 7.2–36) at 3 months and 9.7% (95%CI 3.3–28.4) at 6- and 12 months compared to 96% (95%CI 90.7–100) at 3- and 6 months and 93.9% (95%CI 87.4–100) at 12 months for those of the group 1 (*p* < 0.001). The differences in survival rate were significant in each comparison between the worst group and the others (Fig. 3).

Overall, 97 patients were discharged at home, 39 at rehabilitation centers and 54 in another non-intensive unit of the same hospital or in another hospital of the territory. Discharge disposition differed significantly based on age (p = < 0.001), mFS (*p* < 0.001), BNI grade (*p* = 0.01), quantity of blood in the cistern (*p* = 0.004), the presence of ICH (*p* = 0.028), the group of the SAFIRE or the HATCH score (*p* < 0.001) and the appearance of any complication in the hospital (*p* < 0.001) as well as the permanence in the ICU (*p* < 0.001) or in the ward (*p* < 0.001) (Table 2).

**Table 2:** Frequency table of discharge disposition. Statistic tests: ^1^Linear model Anova; ^2^Pearson’s Chi-squared test; ^3^Trend test for ordinal variables

	Home (*N* = 97)	Rehab (*N* = 39)	Other Hospital (*N* = 54)	Total (*N* = 190)	*p* value
**Age**					< 0.001^1^
Mean (SD)	54.1 (12.8)	61.3 (11.0)	66.7 (12.1)	59.1 (13.4)	
Range	18.0–81.0	38.0–81.0	44.0–87.0	18.0–87.0	
**mFisher**					< 0.001^2^
0	0.0 (0.0%)	0.0 (0.0%)	1.0 (1.9%)	1.0 (0.5%)	
1	17.0 (17.5%)	0.0 (0.0%)	3.0 (5.6%)	20.0 (10.5%)	
2	1.0 (1.0%)	0.0 (0.0%)	1.0 (1.9%)	2.0 (1.1%)	
3	42.0 (43.3%)	11.0 (28.2%)	11.0 (20.4%)	64.0 (33.7%)	
4	37.0 (38.1%)	28.0 (71.8%)	38.0 (70.4%)	103.0 (54.2%)	
**BNI Grade**					0.015^2^
1	0.0 (0.0%)	0.0 (0.0%)	1.0 (1.9%)	1.0 (0.5%)	
2	36.0 (37.1%)	7.0 (17.9%)	14.0 (25.9%)	57.0 (30.0%)	
3	50.0 (51.5%)	22.0 (56.4%)	25.0 (46.3%)	97.0 (51.1%)	
4	10.0 (10.3%)	8.0 (20.5%)	10.0 (18.5%)	28.0 (14.7%)	
5	1.0 (1.0%)	2.0 (5.1%)	4.0 (7.4%)	7.0 (3.7%)	
**Setting After treatment**					< 0.001^3^
Ward	51.0 (52.6%)	3.0 (7.7%)	15.0 (27.8%)	69.0 (36.3%)	
ICU	46.0 (47.4%)	36.0 (92.3%)	39.0 (72.2%)	121.0 (63.7%)	
**LOS in ICU**					< 0.001^1^
N-Miss	51.0	3.0	15.0	69.0	
Mean (SD)	7.9 (10.6)	33.2 (18.1)	27.5 (23.6)	21.7 (20.9)	
Range	1.0–49.0	4.0–108.0	1.0–86.0	1.0–108.0	
**LOS in Ward**					< 0.001^1^
N-Miss	1.0	0.0	9.0	10.0	
Mean (SD)	13.0 (5.9)	28.7 (20.4)	17.4 (14.4)	17.5 (14.0)	
Range	3.0–39.0	4.0–83.0	1.0–60.0	1.0–83.0	
**Complications during Hospitalization**					< 0.001^3^
No	65.0 (67.0%)	2.0 (5.1%)	16.0 (29.6%)	83.0 (43.7%)	
Yes	32.0 (33.0%)	37.0 (94.9%)	38.0 (70.4%)	107.0 (56.3%)	
**SAFIRE Score**					< 0.001^1^
Mean (SD)	4.0 (3.6)	10.2 (4.8)	8.0 (4.1)	6.4 (4.7)	
Range	0.0–14.0	0.0–22.0	0.0–19.0	0.0–22.0	
**SAFIRE Group**					< 0.001^2^
1	43.0 (44.3%)	2.0 (5.1%)	4.0 (7.4%)	49.0 (25.8%)	
2	26.0 (26.8%)	5.0 (12.8%)	14.0 (25.9%)	45.0 (23.7%)	
3	15.0 (15.5%)	6.0 (15.4%)	12.0 (22.2%)	33.0 (17.4%)	
4	13.0 (13.4%)	21.0 (53.8%)	22.0 (40.7%)	56.0 (29.5%)	
5	0.0 (0.0%)	5.0 (12.8%)	2.0 (3.7%)	7.0 (3.7%)	
**HATCH Score**					< 0.001^1^
N-Miss	1.0	0.0	2.0	3.0	
Mean (SD)	2.2 (1.6)	5.6 (1.7)	4.3 (2.0)	3.5 (2.2)	
Range	1.0–8.0	1.0–9.0	1.0–9.0	1.0–9.0	
**HATCH group**					< 0.001^2^
N-Miss	1.0	0.0	2.0	3.0	
1	86.0 (89.6%)	9.0 (23.1%)	31.0 (59.6%)	126.0 (67.4%)	
2	8.0 (8.3%)	20.0 (51.3%)	14.0 (26.9%)	42.0 (22.5%)	
3	2.0 (2.1%)	9.0 (23.1%)	5.0 (9.6%)	16.0 (8.6%)	
4	0.0 (0.0%)	1.0 (2.6%)	2.0 (3.8%)	3.0 (1.6%)	
**HAIR score**					0.324^1^
Mean (SD)	2.4 (2.1)	3.0 (2.2)	2.6 (2.2)	2.6 (2.2)	
Range	0.0–7.0	0.0–6.0	0.0–7.0	0.0–7.0	
**Nutshell (2)**					0.167^1^
Mean (SD)	50.8 (29.2)	61.0 (29.2)	50.9 (31.6)	52.9 (30.0)	
Range	2.7–100.0	5.6–96.5	2.7–99.9	2.7–100.0	

**Fig. 4:**
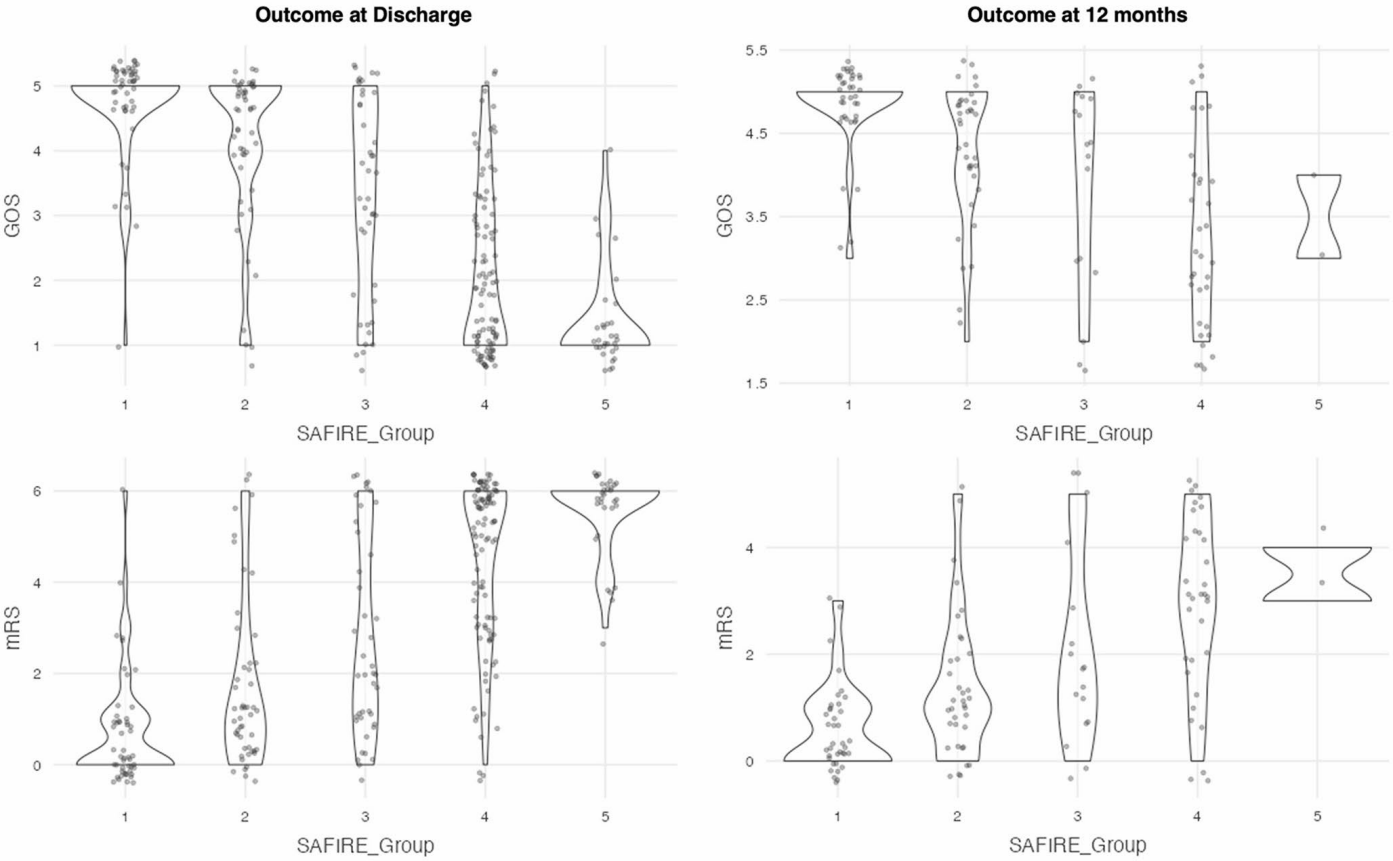
Violin plots of the distribution of patients stratified by outcomes (both modified Rankin Scale and Glasgow Outcome Scale) at discharge and at follow-up (> 6 months) on the y-axis and by SAFIRE groups on the x-axis

A poor outcome was registered in 54.7% of cases at discharge, while for those that reached at least 6 months of follow-up, 28.4% of surviving patients showed a poor outcome (Fig. 4).

Both the SAFIRE and the HATCH scores could predict a poor outcome with a high level of sensitivity and specificity (AUC 0.866 and 0.886, respectively), but it was not the same for the HAIR and the Nutshell (2) (Fig. 2b). Selecting only those patients with more than 12 months of follow-up (126 patients), only the SAFIRE and the HATCH scores reached an adequate AUC value (0.825 and 0.83, respectively) (Fig. 2c).

Considering specifically the worst group of patients among the SAFIRE score (> 15), 31 patients belonged to group 5. Regarding general characteristics, the mean age was 75.2 years old (SD 7.2, range 55.0–91.0), 77.4% were female, 64.5% took a treatment for essential hypertension and almost half of this population had, at least, another cerebral aneurysm. In most cases, the aneurysm was located in the anterior cerebral arteries complex (35.5%) and the mean size was 14.2 mm (SD 8.8, range 3.0–33.0). All patients arrived in the ED in a poor state of consciousness (GCS ≤ 7), almost all were intubated, and 87.1% of patients had blood in the ventricular system. An ICH was diagnosed in 48.4% of cases and 38.7% received no treatment but no variables seemed to show a relationship with this decision, nor the age nor the presence of multiple comorbidities and the clinical status at admission. Clearly, most patients (93.5%) were admitted in ICU and of these 82.8% died in this setting. Out of 31 patients, none were discharged at home, despite a LOS in the hospital of 24.1 days on average (considering both ICU and any ward), 29 died overall on a mean follow-up of 1.6 months (SD 4.2, range 0–19).

Shifting the focus on the group of patients who did not receive any treatment, despite few patients having an acceptable and favorable clinical status at admission, 29 went in the ICU for monitoring and died in this setting and another one died during follow-up. The mean age was 64.1±13.2. Most patients showed a sudden worsening of the neurological functions at presentation: indeed, 59.4% were in GCS 3 and 25% had a sudden cardiac arrest. The decision to avoid any invasive treatment was justified by the absence of brainstem reflexes in 19 patients; brain death appeared secondarily despite EVD placement for 7 patients and in other 6 patients (5 of which with an aneurysm in the posterior circulation) the surgical or the endovascular options were considered not feasible. In the latter situation, 3 patients were admitted in the ward.Only two patients survived at follow-up and had a favorable outcome (mRS 0 and 2). These cases belonged to groups 2 and 3 of the SAFIRE score, which confirmed its association in predicting short-term and long-term outcomes.

## Discussion

Subarachnoid hemorrhage (SAH) remains a severe condition associated with high mortality and unfavorable outcomes. The optimal treatment strategy (coiling vs. clipping) varies based on multiple factors: age, comorbidities, posterior aneurysm location, high mFisher grade, the presence of intracerebral hemorrhage, and worse scores on clinical scales (GCS, H&H, WFNS) play an important role according to the latest guidelines [12]. Additionally, the combination of multiple factors, as per the SAFIRE score, can show an accordance with the treatment strategy as emerged from the current results. Indeed, age (*p* < 0.01), aneurysm size (*p* < 0.01), mFisher grade (*p* < 0.01) and WFNS state after resuscitation (*p* < 0.01) differed significantly between treatment types. These factors can influence the decisional process, firstly, judging between treatment and no treatment and secondly, as elements of discussion for different teams arguing in favor of clipping or coiling.

### Predicting mortality

In line with previous studies [2, 13, 14], the in-hospital mortality rate during the acute phase was 31%, increasing to 38% for patients admitted to ICU. To address this risk, several scoring systems have been developed to predict the severity of the condition, in-hospital mortality [11, 15, 16], and unfavorable long-term outcomes [9, 10, 14, 15].

The current analysis confirms the good performance of the HAIR score in predicting in-hospital death with an AUC of 0.812, consistent with the original study and previous external validation [11, 17]. Although in the latter an overestimation problem was posed, in the studied cohort a higher rate of 30-day mortality has been recognized for lower scores (i.e., 5.8, 14.5 and 17.5 among the first three classes, respectively), facing the opposite problem. However, as the HAIR score was designed to predict in-hospital mortality, its ability to predict long-term outcomes was limited.

Similarly, the web-based AI tool, Nutshell, which uses neural networks, did not demonstrate adequate discrimination for short- and long-term outcome prediction. While it performed moderately well for 30-day mortality prediction (AUC: 0.749), it was inferior to models based on multivariate logistic regression, such as HAIR score. Nonetheless, the tool showed improvement over previous external validations, which reported an AUC of 0.636.

### Predicting the outcome: complications and hospital settings

Approximately 70% of patients experienced complications during hospitalization. The most common neurological complications were vasospasm (clinically and radiologically confirmed), chronic hydrocephalus, and delayed cerebral ischemia (DCI), with frequencies of 20.4%, 19.7%, and 15.0%, respectively. These rates are lower than those reported in the literature for vasospasm and DCI, typically 33% and 20–40% [18, 19], while the appearance of chronic hydrocephalus was in line with previous reports [20]. A statistical significance was observed between the onset of vasospasm and the BNI grade [21].

Although an early treatment (< 24 h) by securing the aneurysm has been associated with reduced mortality and better neurological outcome [12, 22], the subsequent clinical setting can change according to the neurological status and the place where the patient is treated, often depending on bed availability.

Indeed, only 25–30% of patients in “good” neurological status are admitted to non-intensive but highly specialized wards such as Stroke Unit or non-intensive neurosurgical units, decreasing for the former the possibility of offering specialized care for more frequently encountered patients (i.e., ischemic stroke) and for the latter the possibility to offer elective surgery. The same problem becomes exponentially big in the setting of the Intensive Care Unit (ICU) where the cures are highly demanding [23]. On the one hand, admission in a specialized Intensive Care Unit has been demonstrated to offer an advantage on the outcome, by recognizing both neurological and systemic complications that can affect aSAH patients, but, on the other hand, ICU hospitalization may result in additional complications such as ventilator-associated pneumonia, systemic infection or acute kidney injury, which increase the duration and the cost of hospitalization. These factors have been related to a higher risk of delayed medical complications (even in patients with low-grade aSAH), vasospasm, mortality, and 30-day readmissions [24]. Additionally, the duration of ICU stay and mechanical ventilation have been identified as significant contributors to mortality [25].

Similarly to other reports [26], 74.1% of patients were admitted to the ICU, with a mean length of stay of 17.4±19.6 days and a mortality rate of 38.4%. These patients passing through the ICU had a significant (*p* < 0.001) worse outcome (3.9±2.18 for mRS and 2.54±1.54 for GOS) than those who were admitted directly in the ward (1.07±1.48 for mRS and 4.51±0.95 for GOS). Six months has been recognized as the critical period for significant neurological recovery, after which stabilization typically occurs [27]. Indeed, the current population passed from an aggregate mRS of 3.16±2.37 and GOS of 3.05±1.65 at discharge (considering the entire study population) to 1.64±2.17 (mRS) and 4.11±1.30 (GOS) in those with a minimum follow-up of at least 6 months.

### Predicting the outcome: which score?

Evaluating the performance of the SAFIRE and HATCH scores, both demonstrated excellent discriminatory ability for unfavorable outcomes, with AUCs of 0.866 and 0.886 at discharge and 0.825 and 0.83 at one year, respectively. These results confirm the reliability of these scores, with SAFIRE showing improved AUC performance compared to previous validations (AUC: 0.64–0.75) [8, 28, 29].

Among patients with a SAFIRE score > 15 (Group 5) who were treated, an in-hospital mortality rate of 63% was observed, rising to 89.5% if extended to the entire follow-up period (7.2±5.89 months). Overall, a mortality rate of 90% and a poor-outcome rate of 100% were observed (mRS 4 and 5 were reported for the two patients who survived). This supports the evidence that while early mortality may not always be exceedingly high, the risk of poor outcomes increases exponentially in the medium to long term for poor-grade SAH [30]. In this context, prognostic scores such as SAFIRE and HATCH could be particularly valuable.

Indeed, these scores can help identify patients who would require more intensive monitoring despite a relatively stable clinical condition, as well as those who, due to the severity of the SAH, would not benefit from aggressive management in the short and long term. In this regard, these scores also improve communication between physicians and families regarding difficult information. Furthermore, in contexts where the resources are limited, evidence-based data could sustain physicians in choosing the optimal solution to reduce the duration of hospitalization, hospital costs and the suffering of both the patient and the family.

Although the HATCH score demonstrated greater sensitivity and specificity for outcome prediction, it has limitations compared to SAFIRE. For instance, it includes treatment type as a variable, which prevents its use for prognosticating patients in critical condition at diagnosis—precisely when the benefit of treatment is most uncertain, given the high risks of complications, poor outcomes, and extended ICU stays. Moreover, HATCH considers complications such as hydrocephalus, which negatively impact prognosis but may arise long after the initial hemorrhage. Consequently, not all its variables are confined to the admission phase.

The SAFIRE score more than the HATCH score with its simple combination of usually evaluated factors can be readily available at the moment of SAH diagnosis sustaining the decisional process that may take place in the emergency department.

### Limitations

This study presents several limitations that should be acknowledged. Although it was an external validation, the retrospective, single-center design may limit the generalizability of the findings. Indeed, the study’s reliance on historical clinical records and imaging data carries inherent risks of information bias, particularly in evaluating treatment decisions and complications. Additionally, while the study rigorously evaluated the SAFIRE and HATCH scores, other predictive tools and external validations were not comprehensively included, potentially overlooking alternative prognostic indicators. Specifically, the lack of data concerning the SAHIT score is an important limitation but was forced by the impossibility of finding it. Another limitation stems from missing or incomplete follow-up data for some patients, which might have introduced bias in assessing long-term outcomes.

## Conclusions

This study shows how predictive scoring systems, like SAFIRE and HATCH, can be used to manage aSAH. Both tools accurately predicted short- and long-term outcomes, with SAFIRE scoring better for early prognostication. Including prognostic scores in decisions could identify patients unlikely to benefit from aggressive treatment, optimizing resource allocation and reducing ICU burden. In patients with poor-grade SAH, these tools may facilitate communication with families and guide ethical decisions. Future efforts should focus on prospective validation and the development of refined models.

## Electronic supplementary material

Below is the link to the electronic supplementary material.

Supplementary Material 1: Supplementary Table 1: Demographics; clinical and radiological characteristics.
