# Supplementary material for: External validation of outcome prediction scores for aneurysmal subarachnoid hemorrhage in a real-world setting: a monocentric experience
Source: Neurol Sci. 2025 May 17;46(9):4463–74. doi: 10.1007/s10072-025-08232-5 (PMC12394379; doi:10.1007/s10072-025-08232-5)
Supplement: Supplementary file 1 — Supplementary Material 1: Supplementary Table 1: Demographics; clinical and radiological characteristics. [file 10072_2025_8232_MOESM1_ESM.docx]

Supplementary Table 1. Demographics; clinical and radiological characteristics.

|  | **Overall (N=274)** |
| --- | --- |
| **Age** |  |
| Mean (SD) | 60.1 (13.6) |
| Range | 18.0 - 91.0 |
| **Sex** |  |
| Female | 181 (66.1%) |
| Male | 93 (33.9%) |
| **Risk Factors** |  |
| No risk factor | 100 (36.5%) |
| 1 Risk factors | 96 (35.0%) |
| 2 Risk factors | 53 (19.3%) |
| 3 Risk factors | 23 (8.4%) |
| > 3 Risk factors | 2 (0.7%) |
| **Active Smoker** |  |
| No | 212 (77.4%) |
| Yes | 62 (22.6%) |
| **Essential Hypertension** |  |
| No | 141 (51.5%) |
| Yes | 133 (48.5%) |
| **Multiple Aneurysm** |  |
| No | 180 (65.7%) |
| Yes | 94 (34.3%) |
| **How Many Aneurysms?** |  |
| N-Miss | 180 |
| Mean (SD) | 2.5 (0.9) |
| Range | 2.0 - 6.0 |
| **Aneurysm Location** |  |
| ACAs | 109 (39.8%) |
| MCAs | 57 (20.8%) |
| PComAs | 29 (10.6%) |
| ICAs | 35 (12.8%) |
| Posterior Circulation | 44 (16.1%) |
| **Aneurysm** **Size (Dome)** |  |
| Mean (SD) | 8.0 (5.4) |
| Range | 1.5 - 33.0 |
| **GCS at ED** |  |
| 15 | 90 (32.8%) |
| 14 | 33 (12.0%) |
| 13 | 16 (5.8%) |
| 12 | 14 (5.1%) |
| 11 | 3 (1.1%) |
| 10 | 8 (2.9%) |
| 9 | 5 (1.8%) |
| 8 | 8 (2.9%) |
| 7 | 13 (4.7%) |
| 6 | 12 (4.4%) |
| 5 | 10 (3.6%) |
| 4 | 17 (6.2%) |
| 3 | 45 (16.4%) |
| **H&H at ED** |  |
| 1 | 90 (32.8%) |
| 2 | 49 (17.9%) |
| 3 | 27 (9.9%) |
| 4 | 29 (10.6%) |
| 5 | 79 (28.8%) |
| **WFNS at ED** |  |
| 1 | 92 (33.6%) |
| 2 | 35 (12.8%) |
| 3 | 12 (4.4%) |
| 4 | 51 (18.6%) |
| 5 | 84 (30.7%) |
| **mFisher** **Scale** |  |
| 0 | 2 (0.7%) |
| 1 | 20 (7.3%) |
| 2 | 2 (0.7%) |
| 3 | 75 (27.4%) |
| 4 | 175 (63.9%) |
| **BNI Grade** |  |
| 1 | 2 (0.7%) |
| 2 | 63 (23.0%) |
| 3 | 142 (51.8%) |
| 4 | 49 (17.9%) |
| 5 | 18 (6.6%) |
| **Size of SAH (mm)** |  |
| Mean (SD) | 8.1 (3.9) |
| Range | 0.0 - 20.0 |
